# Supplementary material for: The Effect of Massage Force on Relieving Nonspecific Low Back Pain: A Randomized Controlled Trial
Source: Int J Environ Res Public Health. 2022 Oct 13;19(20):13191. doi: 10.3390/ijerph192013191 (PMC9602448; doi:10.3390/ijerph192013191)
Supplement: Supplementary file 1 [file ijerph-19-13191-s001.zip › ijerph-1946636-supplementary.pdf]

Supplementary Table S1. Effects of Massage Force on the Study Outcomes (per protocol)

Variables	HF group	LF group	Mean difference (95% CI)	*p* value[b]	Time *p* value[c]	Time × Group *p* value[c]
Primary outcome						
Pain VAS score in wk 4	2.14 ± 1.49	3.87 ± 1.93	−1.52 (−2.40 to −0.64)	.001		
VAS-MCID in wk 4[a]	17 (68%)	9 (34.6%)		.025		
Secondary outcome						
Pain VAS score					<0.001	.016
Baseline	4.43 ± 1.49	4.76 ± 1.35				
Change in wk 4	−2.40 ± 1.42	−1.04 ± 1.84	−1.36 (−2.29 to -0.43)			
Change in wk 8	−1.57 ± 1.66	−1.55 ± 1.77	−0.15 (−1.00 to 0.97)			
PPT (kg/cm^2) [d]					.098	.038
Baseline	5.52 ± 2.07	5.48 ± 2.26				
Change in wk 4	1.00 ± 1.79	−0.12 ± 1.95	1.15 (0.09 to 2.21)			
Trunk mobility (cm)[d]					.960	.990
Baseline	20.06 ± 1.30	20.40 ±1.01				
Change in wk 4	0.01 ± 1.74	0.01 ± 1.03				
ODI					<0.001	.569
Baseline	18.69 ± 12.90	20.36 ± 13.37				
Change in wk 4	−8.16 ± 9.38	−5.45 ± 13.54				
Change in wk 8	−5.96 ± 11.22	−5.97 ± 10.50				
RMQ					.006	.280
Baseline	7.39 ± 4.95	7.39 ± 4.52				
Change in wk 4	−1.92 ± 3.04	−1.15 ± 5.20				
Change in wk 8	−1.54 ± 3.44	−1.75 ± 4.58				
WHOQOL-physical					<0.001	.470
Baseline	13.35 ± 1.88	13.00 ±2.45				
Change in wk 4	1.38 ± 2.20	0.84 ± 1.75				
Change in wk 8	0.99 ± 1.97	0.87 ± 1.71				
WHOQOL-total					<0.001	.192
Baseline	13.43 ±1.72	13.07 ±1.50				
Change in wk 4	1.15 ± 1.23	0.67 ± 1.27				
Change in wk 8	0.77 ± 1.11	0.25 ± 1.07				

HF, high-force; LF, low-force; PPT; pain pressure threshold; ODI, Oswestry Disability Index; RMQ, Roland–Morris Low Back Pain Disability Questionnaire; Pain VAS, Visual Analog Scale for Pain; WHOQOL-BREF, the short version of the World Health Organization Quality of Life questionnaire.

Values are presented as the mean ± standard deviation or frequency (percentage)

[a] VAS-MCID, the number of participants whose pain reduction reached the minimal clinically important difference on the VAS Pain

[b] Between-group comparison of the primary outcome, either by ANCOVA or chi-square tests.

[c] By repeated measures ANOVA.

[d] PPT and trunk mobility were not evaluated in week 8.
